# Evaluation of factors leading to poor outcomes for pediatric acute lymphoblastic leukemia in Mexico: a multi-institutional report of 2,116 patients

**DOI:** 10.3389/fonc.2023.1255555

**Published:** 2023-09-18

**Authors:** Daniel C. Moreira, Oscar González-Ramella, Maite Echavarría Valenzuela, Angela K. Carrillo, Lane Faughnan, Godwin Job, Yichen Chen, Cesar Villegas, Andrea Ellis Irigoyen, Rosario Barra Urbays, Maribel Ramírez Martinez, Eduardo Altamirano Alvarez, José Antonio León Espitia, Norma Araceli López Facundo, Julia Esther Colunga Pedraza, Flor de María Reyes Gutierrez, Ana Berenice Aguilar Román, Edna Liliana Tamez Gómez, Claudia Selene Portillo Zavala, Natalia del Carmen Negroe Ocampo, Sandra Guadalupe Pulido Sanchez, Deyanira Cortés Alva, Paola Casillas Toral, Karime Salas Villa, Patricia Judith Mendoza Sánchez, Carlos Pérez Alvarado, Gabriela Tamayo Pedraza, Margarita González Zamorano, José Manuel Ricardo Ávila Alba, Jocelyn Becerril Becerril, Hernán Ramírez Durán, Antonio Sandoval Cabrera, Adolfo Pineda Gordillo, Dora Iveth de la Rosa Alonso, Leonardo Javier Mejía Marín, Leslie de los Ángeles Benítez Can, Itzel Gutiérrez Martinez, Mariana Isabel Jiménez Osorio, Naomi Echeandia, Erika Casillas, Karla Guerrero-Gomez, Meenakshi Devidas, Paola Friedrich

**Affiliations:** ^1^ Department of Global Pediatric Medicine, St. Jude Children’s Research Hospital, Memphis, TN, United States; ^2^ Department of Pediatric Hematology/Oncology, Hospital Civil de Guadalajara Juan I. Menchaca, Guadalajara, Mexico; ^3^ Department of Pediatric Oncology, Hospital Pediátrico de Sinaloa, Culiacán, Mexico; ^4^ Department of Pediatric Oncology, Hospital Infantil Teletón de Oncología, Querétaro, Mexico; ^5^ Department of Pediatric Oncology, Centro Estatal de Cancerologia Dr. Miguel Dorantes Mesa, Xalapa, Mexico; ^6^ Department of Pediatric Oncology, Hospital General de Tijuana, Tijuana, Mexico; ^7^ Department of Pediatric Hematology/Oncology, Hospital General con Especialidades “Juan María Salvatierra”, La Paz, Mexico; ^8^ Department of Pediatric Oncology, Hospital General Leoón, León, Mexico; ^9^ Department of Pediatric Oncology, Hospital Materno Infantil ISSEMYM Toluca, Toluca, Mexico; ^10^ Department of Pediatric Hematology, Hospital Universitario “José Eleuterio González”, Monterrey, Mexico; ^11^ Department of Pediatric Hematology/Oncology, Hospital para el Niño del IMIEM, Toluca, Mexico; ^12^ Department of Pediatric Oncology, ONCOCREAN Tapachula, IMSS, Tapachula, Mexico; ^13^ Department of Pediatric Hematology/Oncology, Hospital Infantil de Tamaulipas, Ciudad Victoria, Mexico; ^14^ Department of Pediatric Oncology, Hospital Infantil de Especialidades de Chihuahua, Chihuahua, Mexico; ^15^ Department of Pediatric Oncology, Hospital General Agustín O´Horán, Mérida, Mexico; ^16^ Department of Pediatric Hematology, Hospital Infantil de Morelia “Eva Sámano de López Mateos”, Morelia, Mexico; ^17^ Department of Pediatric Oncology, Hospital del Niño DIF Hidalgo, Pachuca, Mexico; ^18^ Casa de la Amistad, Mexico City, Mexico

**Keywords:** acute lymphoblastic leukemia, Mexico, diagnostic capacity, low-and middle income countries, pediatric oncology

## Abstract

**Background and aims:**

Pediatric acute lymphoblastic leukemia (ALL) survival rates in low- and middle-income countries are lower due to deficiencies in multilevel factors, including access to timely diagnosis, risk-stratified therapy, and comprehensive supportive care. This retrospective study aimed to analyze outcomes for pediatric ALL at 16 centers in Mexico.

**Methods:**

Patients <18 years of age with newly diagnosed B- and T-cell ALL treated between January 2011 and December 2019 were included. Clinical and biological characteristics and their association with outcomes were examined.

**Results:**

Overall, 2,116 patients with a median age of 6.3 years were included. B-cell immunophenotype was identified in 1,889 (89.3%) patients. The median white blood cells at diagnosis were 11.2.5 × 10^3^/mm^3^. CNS-1 status was reported in 1,810 (85.5%), CNS-2 in 67 (3.2%), and CNS-3 in 61 (2.9%). A total of 1,488 patients (70.4%) were classified as high-risk at diagnosis. However, in 52.5% (991/1,889) of patients with B-cell ALL, the reported risk group did not match the calculated risk group allocation based on National Cancer Institute (NCI) criteria. Fluorescence *in situ* hybridization (FISH) and PCR tests were performed for 407 (19.2%) and 736 (34.8%) patients, respectively. Minimal residual disease (MRD) during induction was performed in 1,158 patients (54.7%). The median follow-up was 3.7 years. During induction, 191 patients died (9.1%), and 45 patients (2.1%) experienced induction failure. A total of 365 deaths (17.3%) occurred, including 174 deaths after remission. Six percent (176) of patients abandoned treatment. The 5-year event-free survival (EFS) was 58.9% ± 1.7% for B-cell ALL and 47.4% ± 5.9% for T-cell ALL, while the 5-year overall survival (OS) was 67.5% ± 1.6% for B-cell ALL and 54.3% ± 0.6% for T-cell ALL. The 5-year cumulative incidence of central nervous system (CNS) relapse was 5.5% ± 0.6%. For the whole cohort, significantly higher outcomes were seen for patients aged 1–10 years, with DNA index >0.9, with hyperdiploid ALL, and without substantial treatment modifications. In multivariable analyses, age and Day 15 MRD continued to have a significant effect on EFS.

**Conclusion:**

Outcomes in this multi-institutional cohort describe poor outcomes, influenced by incomplete and inconsistent risk stratification, early toxic death, high on-treatment mortality, and high CNS relapse rate. Adopting comprehensive risk-stratification strategies, evidence-informed de-intensification for favorable-risk patients and optimized supportive care could improve outcomes.

## 1 Introduction

Pediatric acute lymphoblastic leukemia (ALL) is highly curable. Advances in the treatment of ALL embody one of the most successful examples of the progress of the field of pediatric oncology (1). An increased understanding of the biological underpinnings of ALL, the development of risk-stratified treatment, including response-based intensity, and the optimization of supportive care have led to a remarkable increase in cure rates (2, 3). In high-income countries (HICs), survival rates for pediatric ALL have surpassed 90%, and much of the current research focuses on decreasing short- and long-term treatment-related morbidity (4). Nonetheless, the majority of the children diagnosed with ALL live in low- and middle-income countries (LMICs) and do not have access to the optimal care that permits these high cure rates (5). Thus, the success of curing pediatric ALL depends on improving access to quality care for children in LMICs.

Recent studies show that the survival of pediatric patients with ALL varies considerably, with appreciably worse outcomes in LMICs (6). Data from CONCORD-3, an analysis of cancer-related survival from population-based cancer registries, showed survival rates between 50% and 70% for many countries in Latin America, Africa, and Asia (7). A recent simulation-based study estimated the survival of ALL at 61% in Latin America and the Caribbean (6). Mexico is an upper-middle-income country with approximately 2,300 new cases of pediatric ALL each year (8). The estimated 5-year overall survival for ALL in Mexico is approximately 60% (9–11). Adverse outcomes have been associated with late presentation, delayed diagnosis, malnutrition (12), infection-related deaths (13), and abandonment (14). In Mexico, between 2004 and 2019, *Seguro Popular* provided health coverage to the population without social security or private insurance, including financing care for children and adolescents with ALL. Since 2020, the Mexican health system has been in constant redesign. New health governance and financing strategies are being proposed, but their adoption, implementation, spread, and permanence remain to be determined.

In 2016, eight centers from eight different states in Mexico and St. Jude Children’s Research Hospital (St. Jude) in Memphis, United States, joined to create, and in 2017 launch, “Mexico in Alliance with St. Jude (MAS)”, a collaborative group dedicated to increasing the survival and quality of care of children and adolescents with cancer in Mexico. Within the workstream to identify gaps in access and quality care for children with care, a retrospective study was developed to ascertain deficiencies and strengths of care for pediatric ALL. This multi-institutional study sought to characterize the outcomes of children with ALL diagnosed at institutions in Mexico, correlating the findings with clinical and biological factors and elements related to access to quality care. Preliminary results have been used to co-design and co-produce prospective interdisciplinary projects over the years, including an evidence-based, consensus-derived, adapted treatment guideline, which now serves as the standard of care at many MAS member institutions.

## 2 Methods

### 2.1 Study context and oversight

This multicenter retrospective analysis was conducted across 16 pediatric cancer units in Mexico. St. Jude served as a coordinating center for the study, facilitating the electronic database, training for data abstraction, and data analysis. All centers are part of the cooperative group MAS. Institutional review board approval or exemption was obtained at St. Jude and each participating site.

### 2.2 Patient selection and data abstraction

All consecutive patients <18 years of age with newly diagnosed B- and T-cell ALL diagnosed between January 2011 and December 2019 at 16 resource and geographically diverse participating healthcare institutions were included. Clinical information on demographics, treatment, laboratory tests, molecular characteristics, and follow-up was extracted from institutional medical records. For treatment, given that institutions treated ALL with different protocols, and there was variability even within institutions at different timepoints, general descriptors of treatment approach were collected, including chemotherapy doses during induction, use of radiotherapy, and modification to planned treatment. Records were both paper-based and electronic and entered into a single electronic database. The data were first collected in 2018–2019, including inputs through 2015, and then expanded in 2021–2022 to include inputs through 2019 (**Supplement 1**). Data collection was completed in December of 2022.

### 2.3 Statistical analyses

Descriptive statistics were used to summarize patient characteristics. Categorical data are presented as percentages, and continuous data as means (standard deviations) or medians (interquartile range (IQR)). Event-free survival (EFS) and overall survival (OS) were calculated by the Kaplan–Meier method with standard errors by Peto et al. (15, 16) EFS was defined as the time from diagnosis to first event (induction failure, induction death, relapse, and remission death) or date of last contact for those who were event-free. OS was defined as the time from diagnosis to death or last contact for those still alive. For abandonment-sensitive EFS (A-EFS) and OS (A-OS), treatment abandonment was also considered an event. Log-rank test was used to compare survival curves between groups. Cumulative incidence rates were computed using the cumulative incidence function for competing risks, and comparisons were made using the *K*-sample test (17). Univariate and multivariable Cox regression analyses were used to assess the effect of factors on EFS. For all analyses, a p-value <0.05 was considered statistically significant. Analyses were conducted using SAS software, version 9.4, and R version 4.0.0.

## 3 Results

A total of 2,116 eligible patients were identified with a median age of 6.3 years (IQR, 7.5). Patient characteristics are presented in **Table 1**. Most patients had B-cell ALL (1,889, 89.3%) and no central nervous system (CNS) involvement (1,810, 85.5%). Only 160 (7.6%) of patients presented with T-cell phenotype, and only 61 (2.9%) of the patients were reported to have trisomy 21 (Down syndrome). The diagnostic lumbar puncture was traumatic in 127 patients (6.0%). Data on CNS status, immunophenotype, and karyotype were not available in 130 (6.1%), 39 (1.8%), and 745 (35.2%) patients, respectively.

**Table 1:** Patient characteristics.

Characteristic	Value, n (%)
Treatment hospital city	
*Guadalajara*	419 (19.8)
*Toluca*	368 (17.4)
*Pachuca*	282 (13.3)
*Culiacán*	170 (8.0)
*Toluca 2*	129 (6.1)
*Xalapa*	120 (5.7)
*Monterrey*	116 (5.5)
*Tijuana*	114 (5.4)
*Querétaro*	86 (4.1)
*León*	68 (3.2)
*Mérida*	68 (3.2)
*Morelia*	59 (2.8)
*Tapachula*	35 (1.7)
*La Paz*	34 (1.6)
*Ciudad Victoria*	27 (1.3)
*Chihuahua*	21 (1.0)
Sex *(n, %)*	
*Male*	1,188 (56.1)
Age (years)	
*Median (SD)*	6.3 (4.6)
Age category (years)	
<1	59 (2.8)
1–10	1,430 (67.6)
≥10	627 (29.6)
WBC at diagnosis (×10^3^/mm^3^)	
*Median (SD)*	11.2 (110.7)
CNS status	
*CNS-1*	1,810 (85.5)
*CNS-2*	67 (3.2)
*CNS-3*	61 (2.9)
*Not evaluated*	47 (2.2)
*Result not available*	130 (6.1)
Immunophenotype	
*B cell*	1,889 (89.3)
*T cell*	160 (7.6)
*Test sent, not interpretable*	17 (0.8)
*Test not sent/requested*	11 (0.5)
*Result not available*	39 (1.8)
Karyotype	
46	460 (21.7)
≤45	26 (1.2)
47–50	46 (2.2)
>50	82 (3.9)
*Test sent, not interpretable*	203 (9.6)
*Test not sent/requested*	554 (26.2)
*Result not available*	745 (35.2)
Trisomy 21	
*No*	2,055 (97.1)
*Yes*	61 (2.9)

WBC, white blood cell; CNS, central nervous system.

### 3.1 Risk stratification and treatment

Of the 1,889 patients with B-cell ALL, 1,488 (70.4%) patients were assigned high-risk therapy at the beginning of treatment, while 593 (28.0%) were assigned standard-risk therapy. Based on National Cancer Institute (NCI) standard-risk criteria for pediatric B-cell ALL (age 1–10 years and initial white blood cell (WBC) <50,000/mm^3^), 52.5% (991/1,889) of patients were assigned a risk group at the start of treatment that differs from what would be obtained utilizing this standard. Distribution of age and initial WBC shows that only 28.5% (538/1,889) and 23.2% (439/1,889) of patients, respectively, would fall within high-risk criteria for these two parameters. After induction therapy, patients were assigned final risk groups: 447 patients (23.3%) were classified as standard-risk, 1,262 patients (65.9%) as high-risk, and 126 patients (6.6%) as very high-risk. Risk-stratification data were unavailable for 200 patients.

Regarding treatment, 148 patients (7.0%) had received treatment for ALL prior to transfer to one of the 16 institutions, with the majority being the initiation of systemic corticosteroids. The 16 institutions used treatment protocols adapted from BFM, St. Jude’s Total XIIIB, or the Dana-Farber Cancer Institute (DFCI) 05-001 protocols. Prednisone, vincristine, asparaginase, and daunorubicin were the most used systemic chemotherapy agents during induction. The median doxorubicin equivalents during induction were 60 mg/m^2^ (IQR, 25.0). The median number of intrathecal chemotherapy doses was 4.0 (IQR, 3.0), with triple therapy (methotrexate, cytarabine, and hydrocortisone) being the most used intrathecal chemotherapy (71.1%). Among the 74 patients with BCR-ABL translocation, 24 (32.4%) started imatinib during induction. Sixty patients (3.1%) received radiation as part of treatment: 34 (56.7%) with CNS-1 status, 6 (10%) with CNS-2, 17 (28.3%) with CNS-3, and 3 (5%) with unknown CNS status.

During therapy, 323 patients (15.3%) had a substantial change to treatment, defined as the elimination or substitution of a chemotherapy agent in more than half of the doses of a treatment phase. Modifications due to toxicity or infection were the most frequently cited reason (38.1%, 123/323). Furthermore, the mean number of times chemotherapy was held for more than 2 weeks was 1.5 times per patient. Infection (68.7%) and chemotherapy-related side effects (56.8%) were the most cited reasons for the interruption of chemotherapy.

### 3.2 Access to molecular diagnostics

Fluorescence *in situ* hybridization (FISH) and PCR tests were performed for 407 (19.2%) and 736 (34.8%) patients, respectively. The median times to obtain results for the characterization of ALL samples were as follows: 1.0 days for immunophenotype (standard deviation (SD), 6.8 days), 10.0 days for FISH (SD, 13.7 days), 10.0 days for cytogenetics (SD, 23.0 days), and 8.0 days for PCR (SD, 14.8 days). To evaluate the availability of molecular tests and the frequency of recurrent alterations, five common translocations were assessed in patients with B-cell ALL (**Table 2**). Considering the cases where these tests were performed and available, samples were positive in 10.8% (106/981) for ETV6-RUNX1, 6.5% (74/1,133) for BCR-ABL, 7.8% (74/953) for E2A/PBX, 4.3% (40/940) for MLL translocations, and 6.4% (14/220) for iAMP21.

**Table 2:** Testing of recurrent translocations for B-cell ALL.

Characteristic	ETV6-RUNX/t(12,21) n (%)	BCR-ABL/t(9,22) n (%)	E2A/PBX/t(1,19) n (%)	MLL (4,11) n (%)	iAMP21 n(%)
Not done	716 (33.8)	566 (26.7)	786 (37.1)	796 (37.6)	1,472 (69.6)
Negative	872 (41.2)	1,054 (49.8)	873 (41.3)	894 (42.2)	200 (9.5)
Positive	106 (5.0)	74 (3.5)	74 (3.5)	40 (1.9)	14 (0.7)
Not available	419 (19.8)	417 (19.7)	377 (17.8)	380 (18.0)	424 (20.0)
Not interpretable	3 (0.1)	5 (0.2)	6 (0.3)	6 (0.3)	6 (0.3)

ALL, acute lymphoblastic leukemia.

Minimal residual disease (MRD) was performed in 1,158 patients (54.7%) during induction (**Table 3**). All MRD tests were performed from bone marrow aspirate samples. Days 15 (35.5%) and 29 (29.1%) were the most common timepoints for MRD evaluation. Considering negative MRD thresholds of <1% on Days 8 and 15 and <0.01% on Day 29, 87.1% (27/31), 84.8% (341/402), and 73.9% (235/318) of tested patients had negative MRD during these timepoints, respectively.

**Table 3:** MRD monitoring for B-cell ALL.

	MRD D8 n (%)	MRD D15 n (%)	MRD D29 n (%)	MRD D42 n (%)	MRD Other n (%)
**Tested**	31 (2.7)	411 (35.5)	337 (29.1)	44 (3.8)	335 (15.2)
*<0.01%*	24 (77.4)	282 (68.6)	235 (69.7)	24 (54.6)	216 (64.5)
*0.01%–0.99%*	3 (9.7)	59 (14.4)	57 (16.9)	12 (27.3)	66 (19.7)
*≥1%*	4 (12.9)	59 (14.4)	23 (6.8)	2 (4.6)	37 (22.0)
*Sent, not interpretable*	0 (0)	2 (0.5)	19 (5.6)	6 (13.6)	15 (4.5)
*Data not found*	0 (0)	9 (2.2)	3 (0.9)	0 (0)	1 (0.3)

MRD, minimal residual disease; ALL, acute lymphoblastic leukemia.

### 3.3 Early toxicity and death

During induction, there were 191 deaths (9.1%) reported: 142 in patients with B-cell ALL (7.5% of B-cell ALL patients) and 49 in patients with T-cell ALL (30.1% of T-cell ALL). Antibiotics were indicated for therapeutic purposes during induction in 1,536 patients (72.6%). Finally, clinical sepsis (542 patients), bacteremia (487 patients), pneumonia (276 patients), and meningitis (9 patients) were the most reported infections during induction.

### 3.4 Outcomes

Median follow-up was 3.7 years and was available for 2,106 patients (99.5%). Forty-five patients (2.1%) had induction failure. A total of 365 deaths (17.3%) were reported, including 191 deaths during induction and 174 additional deaths after achieving remission. Of the patients, 176 (6%) abandoned treatment, and 416 (19.6%) relapsed. Among the relapses, 225 (54.1%) were isolated bone marrow, 111 (26.7%) isolated CNS, 63 (15.1%) mixed, and 17 (4.1%) extramedullary (non-CNS) relapses.

**Figure 1:**
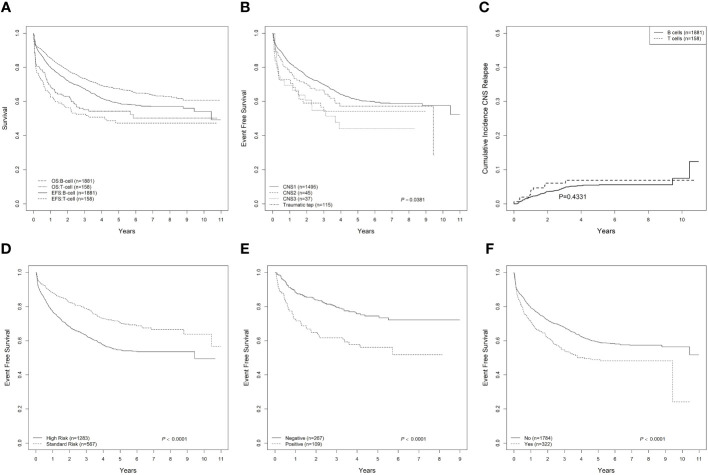
Outcome of pediatric ALL in Mexico. **(A)** EFS and OS for B-cell and T-cell ALL. **(B)** EFS for CNS status for B-cell ALL. **(C)** Cumulative incidence of CNS relapse for B-cell and T-cell ALL. **(D)** EFS for standard-risk and high-risk B-cell ALL. **(E)** EFS for B-cell ALL based on MRD status tested on Day 15. **(F)** EFS for B-cell and T-cell ALL based on substantial change to treatment. ALL, acute lymphoblastic leukemia; EFS, event-free survival; OS, overall survival; CNS, central nervous system; MRD, minimal residual disease.

Five-year EFS and OS rates based on clinical and biological characteristics are given in **Table 4** and **Figure 1**. For the whole cohort, the 5-year EFS and OS were 57.6% ± 1.6% and 66.0%± 1.5%, respectively. The 5-year A-EFS and A-OS for the whole cohort were 54.5% ± 1.6% and 62.1% ± 1.5, respectively. The 5-year EFS was 58.9% ± 1.7% for B-cell ALL and 47.4% ± 5.9% for T-cell ALL, while the 5-year OS was 67.5% ± 1.6% for B-cell ALL and 54.3% ± 5.9% for T-cell ALL (**Figure 1A**). For the whole cohort, significantly higher outcomes were observed for patients aged 1–10 years and patients without substantial treatment modifications. Outcomes from the geographic territories with the highest number of patients are included in **Supplement 2**.

**Table 4:** EFS and OS by clinical and biological characteristics.

Characteristic	No. of patients	5-Year EFS% (95% CI)	p	5-Year OS% (95% CI)	p
All patients					
*Overall EFS and OS*	2,106	57.6 ± 1.6		66.0 ± 1.5	
*Abandonment-sensitive EFS and OS*	2,106	54.5 ± 1.6		62.1 ± 1.5	
Age					
<1 year	59	26.7 ± 9.3	<0.0001	30.3 ± 8.8	<0.0001
1–10 years	1,422	64.1 ± 1.8		74.3 ± 1.6	
>10 years	625	45.4 ± 3.3		49.8 ± 3.2	
*Trisomy 21*	61	37.7 ± 9.4	–	53.8 ± 10.6	
MRD performed					
*Yes*	1,151	66.3 ± 2.4	<0.0001	75.3 ± 2.2	<0.0001
*No*	955	47.6 ± 2.0		55.3 ± 2.0	
Treatment modification					
No substantial change	1,784	59.2 ± 16.9	<0.0001	68.0 ± 1.6	<0.0001
Substantial change	322	49.0 ± 4.3		55.3 ± 4.4	
B-cell ALL					
*All B-cell*	1,881	58.9 ± 1.7		67.5 ± 1.6	
CNS status					
CNS-1	1,495	61.1 ± 1.8	0.038	69.6 ± 1.7	0.34
CNS-2	45	54.1 ± 11.1		67.2 ± 10.3	
CNS-3	37	44.0 ± 10.4		58.7 ± 10.4	
Traumatic	11e5	57.3 ± 8.4		64.6 ± 7.9	
WBC at diagnosis					
<50,000/mm^3^	1,556	62.9 ± 1.8	<0.0001	70.9 ± 1.8	<0.0001
≥50–100,000/mm^3^	467	42.3 ± 3.7		53.2 ± 3.8	
Initial risk group					
Standard risk	591	70.5 ± 2.8	<0.0001	79.8 ± 2.5	<0.0001
High risk	1,480	54.6 ± 2.0		62.9 ± 2.0	
Translocations					
t(9;22)(BCR-ABL1)	71	40.5 ± 10.4	<0.0001	45.6 ± 9.7	<0.0001
t(12;21)(ETV6-RUNX1)	104	72.2 ± 8.1		81.2 ± 6.8	
t(1;19)(E2A-PBX1)	69	68.9 ± 9.6		71.1 ± 9.0	
KMT2A rearrangement	38	33.2 ± 12.1		38.1 ± 13.4	
iAMP21	14	70.9 ± 19.1		67.5 ± 19.2	
Ploidy					
>50 chromosomes	82	84.4 ± 5.6	0.0046	91.4 ± 4.3	0.0085
≤50 chromosomes	490	65.0 ± 3.3		74.6 ± 3.0	
MRD Day 15					
Negative	267	74.5 ± 4.3	<0.0001	79.2 ± 3.0	0.0002
Positive	109	56.2 ± 9.3		64.4 ± 8.6	
MRD Day 29					
Negative	216	75.7 ± 5.1	0.093	83.4 ± 4.3	0.254
Positive	72	66.6 ± 7.9		80.0 ± 6.6	
T-cell ALL					
*All T-cell*	158	47.4 ± 5.9		54.3 ± 5.9	
CNS status					
CNS-1	107	51.8 ± 6.8	0.85	60.5 ± 6.7	0.73
CNS-2	4	50.0 ± 35.4		50.0 ± 35.4	
CNS-3	13	50.8 ± 20.6		50.8 ± 20.6	
Traumatic	9	64.8 ± 38.5		64.8 ± 38.5	

EFS, event-free survival; OS, overall survival; MRD, minimal residual disease; CNS, central nervous system; WBC, white blood cell; ALL, acute lymphoblastic leukemia.

Translocations also showed varying outcomes for B-cell ALL, with patients with t(9;22) (BCR-ABL1) having a 5-year EFS of 40.5% ± 10.4%. The 5-year EFS for B-cell ALL with CNS-1, CNS-2, and CNS-3 was 61.1% ± 1.8%, 54.1% ± 11.1%, and 44.0% ± 10.4%, respectively (p = 0.038) (**Figure 1B**). The 5-year cumulative incidence of CNS relapse was 5.5% ± 0.6% (**Figure 1C**). The 5-year EFS for B-cell ALL was 70.5% ± 2.8% for the standard-risk group and 54.6% ± 2.0% for the high-risk group (p < 0.0001) (**Figure 1D**). In patients with B-cell ALL and MRD performed on Day 15, the 5-year EFS was 74.5% ± 4.3% for patients with negative MRD and 56.2% ± 9.3% for patients with positive MRD (**Figure 1E**, p<0.001). For patients with substantial modifications to treatment (**Figure 1F**), 5-year EFS was also lower (p < 0.0001).

Univariate and multivariable Cox regression analyses of EFS were performed for B-ALL (**Table 5**). In univariate analyses, age (1–10 years), initial risk group (standard risk), and Day 15 MRD (negative) were significantly associated with lower EFS. In multivariable analyses, age and Day 15 MRD continued to have a significant effect on EFS.

**Table 5:** Univariate and multivariable Cox regression analyses of EFS for B-cell ALL limited to patients with Day 15 MRD data.

Effect	Univariate analysis			Multivariable analysis (N = 367)	
	N	Hazard ratio	p-value	Hazard ratio	p-value
Age			**0.0013**		**0.0064**
*<1*	10	2.19 (0.80–6.02)		1.93 (0.69–5.34)	
*1–10*	264	Ref		Ref	
*≥10*	111	1.97 (1.34–2.89)		1.91 (1.27–2.87)	
Sex			0.4770		
*Male*	206	0.87 (0.60–1.27)			
*Female*	179	Ref			
WBC (×10^3^/mm^3^)			0.4733		
<50	296	Ref			
≥50	74	1.18 (0.75–1.86)			
CNS status			0.2824		
*CNS-1*	315	Ref			
*CNS-2*	7	0.45 (0.06–3.24)			
*CNS-3*	13	1.75 (0.76–4.00)			
Molecular biology			0.8230		
*Favorable*	16	Ref			
*Neutral*	60	1.28 (0.37–4.41)			
*Unfavorable*	29	1.50 (0.41–5.53)			
Initial risk group			**0.0103**		0.0992
*Standard*	82	Ref		Ref	
*High*	294	2.15 (1.18–3.93)		1.73 (0.90–3.32)	
Final risk group			0.0813		
*Standard*	68	Ref			
*High*	272	1.75 (0.93–3.28)			
*Very High*	10	3.26 (1.04–10.23)			
MRD Day 15 status			**<0.0001**		**<0.0001**
*Positive*	109	2.15 (1.47–3.15)		2.16 (1.47–3.18)	
*Negative*	267	Ref		Ref	

EFS, event-free survival; ALL, acute lymphoblastic leukemia; MRD, minimal residual disease; WBC, white blood cell; CNS, central nervous system.

Bold values are those that have a p value of less than 0.05.

## 4 Discussion

This detailed review of a large cohort of patients diagnosed and treated in Mexico has allowed for the evaluation of multiple elements of pediatric ALL care and outcomes. Our study analyzed data from 2,116 patients across 16 centers and suggests outcomes are lower than desirable, with a high frequency of treatment-related toxicity and relapse, and inconsistent access to diagnostic tests, both at diagnosis to characterize ALL samples and for disease monitoring through MRD.

Consistent with prior institutional and population-based cancer registry reports from Mexico (7, 9–11), our study describes lower OS and EFS for pediatric ALL than reported in HICs. Furthermore, regional variability can also be seen in our study, similar to published reports (9). Compared to reports from collaborative studies from North America and Europe, the outcomes from our report are close to 20% to 30% lower. High rates of death in induction, abandonment, death in remission, and relapse are the main contributing factors to these poor outcomes. Nonetheless, factors associated with outcomes from high-resource settings such as age, WBC count at diagnosis, ploidy, presence of translocation, and response to therapy (MRD) continue to portend prognostic significance.

We report an abandonment rate of 6%, like prior reports from Mexico (10, 18, 19) During the time that this cohort was treated, government-funded healthcare existed for children and adolescents with ALL. Despite coverage, treatment abandonment is a complex phenomenon, and it has been associated with social, economic, and treatment-related factors (20, 21). Social inequalities and social determinants of health continue to impact cancer outcomes even when direct costs for cancer care are covered (22). This study did not collect data on social determinants of health for the cohort; hence, additional analyses would be necessary to further describe factors that increase the risk of treatment abandonment in Mexico.

Induction has the highest risk of infectious complications due to prolonged immunosuppression from disease and the intensity of induction chemotherapy (23, 24). In our study, close to 10% mortality was seen during induction, with most deaths from infectious processes. This rate is markedly above reports not only of the clinical trials of cooperative groups in high-resource settings where <2% is usually reported (2, 25, 26) but also in pediatric oncology units in Central America (27, 28). The risk of death in induction was not associated with treatment intensity (anthracycline dose), suggesting that earlier diagnosis of ALL and improved management of infectious complications may be the most valuable intervention. Importantly, the MAS centers are participating in a project to implement a pediatric early warning system (PEWS) (29) and timely antibiotic administration in febrile patients (30) as mechanisms to improve treatment-related mortality. Additional interventions such as prophylactic antibiotics during induction (31) could be considered as strategies to decrease infectious mortality early in treatment. Interventions that mitigate unplanned toxicities could also impact the financial aspects of pediatric cancer care (32).

Contrary to most pediatric ALL studies, our study reports that most patients were classified as high-risk, including 70% at the beginning of induction. The NCI standard-risk criteria are effective risk stratification criteria (33), especially when access to molecular tests is inconsistent. Based on these two variables, we describe how close to 50% of patients were inappropriately assigned risk groups. Although other variables are used for risk assignment, such as steroid response and DNA index, this is unlikely to explain the large disparity. Ultimately, these data suggest that a large proportion of patients were over-treated, increasing the risk of treatment-related mortality and long-term morbidity. This is especially worrisome, as it is known that close to 40% of patients can be cured with minimally intensive therapy (34, 35). Standardized approach to risk stratification would optimize the intensity of therapy based on the risk of relapse.

In our report, the frequency of CNS-2 status was 3.2%. This is lower than recently published studies from North America, where St. Jude’s Total XVI had 33% (24) and COG’s standard-risk trial, AALL0331, had 8% (25). On further investigation, some centers included in the study did not have the capacity to perform cytospin to evaluate for leukemic blasts, hence likely under-reporting CNS-2 status. Given these elements and the previously mentioned discordance in risk stratification, the association of CNS status and outcomes in this cohort was likely confounded due to these factors.

Over the past decades, the molecular characterization of ALL has transformed the field of pediatric leukemia care (36). Access to advanced diagnostic tests to characterize ALL samples are essential to provide risk-adapted therapy, seeking to maximize cure rates and minimize therapy-associated toxicities. Our data suggested limited access to advanced diagnostics like FISH and PCR. Furthermore, uninterpretable tests were also reported, highlighting the need to improve the quality of testing also. Strategies to achieve increased access to quality diagnostics could be the identification of centralized, regional testing centers with adequate validation processes.

Response to treatment and MRD has been the most important prognostic factor for pediatric ALL (37–40). The role of MRD in risk stratification has already been used in LMICs for close to two decades, confirming its relevance in resource-limited settings even when simplified flow cytometry-based assays are utilized (41, 42). In our study, 55% of patients had MRD performed during different points of therapy. Importantly, in univariate and multivariable analyses, negative MRD was associated with EFS. Furthermore, patients whose MRD was performed, regardless of results, had better survival, suggesting that access to comprehensive testing can influence outcomes. Based on these data, consistent access to timely and high-quality MRD would optimize therapy for patients with ALL. Increasing access to MRD could be achieved by identifying regional centers to process samples for treatment response.

Pediatric ALL is a heterogeneous disease, with recurrent genetic alterations conferring treatment prognosis. Some studies have described a lower frequency of favorable translocations, like ETV6-RUNX1, in Hispanic populations within the United States (43). The frequency of ETV6-RUNX1 in our cohort is consistent with Hispanic patients in the United States, which is lower than other populations. Of the patients with ALL and BCR-ABL fusions, the 5-year OS was 45.6%, consistent with the outcomes seen before the use of ABL-class tyrosine kinase inhibitors (44). In our study, of the 74 patients identified with BCR-ABL1 fusions, only 24 received targeted therapy, likely hindering the possibility of early remission. It is important to note that an important fraction of the cohort does not have alterations frequently seen in pediatric ALL. It is unclear if this is related to a true variation or inherently a marker of inadequate access to comprehensive, validated molecular testing. Ultimately, the size of the cohort with complete molecular characterizations is insufficient to comprehensively describe variations of genetic alterations in ALL for the Mexican population.

Our study has limitations. As a retrospective study, the availability of all details of care was absent for some patients, especially as we sought to extract granular features of diagnostic evaluations and therapy. Nonetheless, given the size of the cohort and explicit mention of when data elements were absent, relevant conclusions can still be reached. Furthermore, as patients were treated with different protocols, the impact of specific treatment phases and chemotherapy strategies cannot be concluded from this study.

In Mexico, cancer is the leading cause of death in children aged 5–14 (45, 46); hence, investment in the care of children with ALL and pediatric cancer is imperative. The results from this study highlight areas that are relevant for interventions to improve quality care for children with ALL not only in Mexico but also in other LMICs. Based on these data, the MAS group has developed an evidence-based consensus-derived treatment guideline for ALL currently being used in more than 10 pediatric cancer units in Mexico. Furthermore, these data informed a peer-reviewed grant-funded prospective project to improve access to a consensus-derived diagnostic panel and support comprehensive risk stratification. Some of the outputs of these interventions are included in other manuscripts of this *Frontiers in Oncology Research Topic*. With these data-driven approaches, improved outcomes are anticipated.

## Data availability statement

The raw data supporting the conclusions of this article will be made available by the authors, without undue reservation.

## Ethics statement

The studies involving humans were approved by St. Jude Children’s Research Hospital and each participating site. The studies were conducted in accordance with the local legislation and institutional requirements. Written informed consent for participation was not required from the participants or the participants’ legal guardians/next of kin. Exemption is due to retrospective nature of study.

## Author contributions

DM: Formal Analysis, Supervision, Visualization, Writing – original draft, Writing – review & editing. OG-R: Investigation, Supervision, Writing – review & editing. ME: Investigation, Supervision, Writing – review & editing. AC: Data curation, Project administration, Writing – review & editing. LF: Project administration, Writing – review & editing. GJ: Formal Analysis, Writing – review & editing. YC: Formal Analysis, Writing – review & editing. CV: Supervision, Writing – review & editing. AI: Investigation, Writing – review & editing. RB: Investigation, Writing – review & editing. MR: Data curation, Investigation, Writing – review & editing. EA: Investigation, Writing – review & editing. JE: Investigation, Writing – review & editing. NL: Investigation, Writing – review & editing. JC: Investigation, Writing – review & editing. FR: Investigation, Writing – review & editing. AA: Investigation, Writing – review & editing. ET: Investigation, Writing – review & editing. CPZ: Investigation, Writing – review & editing. NN: Investigation, Writing – review & editing. SP: Investigation, Writing – review & editing. DC: Investigation, Writing – review & editing. PC: Data curation, Writing – review & editing. KS: Data curation, Writing – review & editing. PM: Data curation, Writing – review & editing. CPA: Data curation, Writing – review & editing. GT: Data curation, Writing – review & editing. MG: Data curation, Writing – review & editing. JÁ: Data curation, Writing – review & editing. JB: Data curation, Writing – review & editing. HR: Data curation, Writing – review & editing. AS: Data curation, Writing – review & editing. AP: Data curation, Writing – review & editing. DA: Data curation, Writing – review & editing. LM: Data curation, Writing – review & editing. LB: Data curation, Writing – review & editing. IG: Data curation, Writing – review & editing. MJ: Data curation, Writing – review & editing. NE: Data curation, Writing – review & editing. EC: Data curation, Supervision, Writing – review & editing. KG: Supervision, Writing – review & editing. MD: Formal Analysis, Writing – review & editing. PF: Conceptualization, Investigation, Methodology, Project administration, Resources, Writing – review & editing.
