# Supplementary material for: Evaluation of factors leading to poor outcomes for pediatric acute lymphoblastic leukemia in Mexico: a multi-institutional report of 2,116 patients
Source: Front Oncol. 2023 Sep 18;13:1255555. doi: 10.3389/fonc.2023.1255555 (PMC10544893; doi:10.3389/fonc.2023.1255555)
Supplement: Supplementary file 1 [file Table_1.docx]

Supplement 1: Time intervals of collected data by institution.

| **2011-2019** | **2016-2019** | **2011-2016** |
| --- | --- | --- |
| - Guadalajara - Toluca - Culiacán - Pachuca - Tijuana - Querétaro - Mérida - Tapachula - La Paz - Chihuahua | - Monterrey - Toluca 2 - León - Morelia - Ciudad Victoria | - Xalapa |
