# Supplementary material for: Evaluation of factors leading to poor outcomes for pediatric acute lymphoblastic leukemia in Mexico: a multi-institutional report of 2,116 patients
Source: Front Oncol. 2023 Sep 18;13:1255555. doi: 10.3389/fonc.2023.1255555 (PMC10544893; doi:10.3389/fonc.2023.1255555)
Supplement: Supplementary file 2 [file Table_2.docx]

Supplement 2: Analysis of outcomes in the 4 states contributing the most patients.

| **State** | **No. of Patients** | **5-Year OS% (95% CI)** | ***P*** |
| --- | --- | --- | --- |
| Estado de México | 496 | 64.3±2.9 | p=0.0001 |
| Jalisco | 413 | 65.8±3.4 |  |
| Hidalgo | 282 | 50.7±3.8 |  |
| Sinaloa | 170 | 67.7±4.9 |  |
